# Roles of interleukin-11 during acute bacterial pneumonia

**DOI:** 10.1371/journal.pone.0221029

**Published:** 2019-08-15

**Authors:** Katrina E. Traber, Ernest L. Dimbo, Elise M. Symer, Filiz T. Korkmaz, Matthew R. Jones, Joseph P. Mizgerd, Lee J. Quinton

**Affiliations:** 1 Pulmonary Center, Boston University School of Medicine, Boston, Massachusetts, United States of America; 2 Department of Medicine, Boston University School of Medicine, Boston, Massachusetts, United States of America; 3 Department of Microbiology, Boston University School of Medicine, Boston, Massachusetts, United States of America; 4 Department of Biochemistry, Boston University School of Medicine, Boston, Massachusetts, United States of America; 5 Department of Pathology and Laboratory Medicine, Boston University School of Medicine, Boston, Massachusetts, United States of America; Forschungszentrum Borstel Leibniz-Zentrum fur Medizin und Biowissenschaften, GERMANY

## Abstract

Interleukin-11 (IL-11) is an interleukin-6 (IL-6) family cytokine shown to play a protective role in acute inflammatory settings including systemic infection. In this study we addressed the role of IL-11 in acute bacterial pneumonia using a mouse model of *E*. *coli* pneumonia. Compared with other related cytokines, IL-11 protein was maintained at high levels in the lung at baseline, with only mild alterations in whole lung and BALF levels during acute infection. The primary source of IL-11 in the lung was the epithelium, but steady state production was not dependent on the inflammatory transcription factor nuclear factor kappa B in cells of either myeloid or epithelial lineage. Blockade of IL-11 with neutralizing antibodies resulted in a mild but significant decrease in neutrophil recruitment and increase in pulmonary edema during pneumonia, without detectable alterations in bacterial clearance. Exogenous IL-11 administration, however, had no effect at baseline or during infection. Overall, we conclude that maintenance of lung IL-11 concentrations may influence acute pulmonary inflammation during infection, albeit modestly.

## Introduction

Pneumonia and influenza together represent the leading infectious cause of death in the US [1], the fifth leading cause of death worldwide, and a substantial cause of loss of productive life years due to morbidity [2]. The current standard of care for pneumonia is centered on microbial killing and/or neutralization, plus generalized supportive care. However, it is becoming increasingly clear that a significant portion of the injury caused by pneumonia is driven by immunopathology, and the ongoing emergence of antibiotic-resistant pathogens further reinforces the need to investigate biological systems controlling both immune resistance and host tissue resilience. Ultimately, a better understanding of the signaling pathways controlling innate immunity is essential for the discovery of novel clinical interventions [3].

The IL-6 family of cytokines is a pleiotropic group of factors including IL-6, IL-11, IL-27, IL-31, Leukemia Inhibitory Factor (LIF), and Oncostatin M (OSM) [4, 5]. Signaling from these cytokines involves numerous receptors, but all share a requirement for the signal-transducing β-receptor subunit glycoprotein 130 (gp130) [6]. Similar to the canonical IL-6 family member, but unlike other members of this group, IL-11 signals by first binding a non-signaling α-receptor, IL-11Rα, along with gp130. Subsequent dimerization generates a hexameric signaling complex, which ultimately leads to JAK mediated STAT3 activation [6]. IL-11 has been known for some time to play a role in hematopoiesis, and can be used clinically for the treatment of chemotherapy-induced thrombocytopenia [7]. In addition to its role in hematopoiesis, IL-11 contributions have also been implicated during inflammation, but its specific role has been elusive. It appears that in many cases, IL-11 has an anti-inflammatory effect, limiting inflammatory cytokine production [8–10], without limiting host defense [11, 12]. However, sustained production of IL-11 can lead to lymphocytic and eosinophilic inflammation, and can ultimately promote fibrosis [13–16].

Under baseline homeostatic conditions, IL-11 is not usually detected in the circulation, but has been shown to locally increase at sites of inflammation [17, 18]. Our previous work demonstrated a small increase in IL-11 mRNA in the lung during pneumonia, suggesting a possible role for this cytokine during infection [19]. In addition, several members of the IL-6 family play disparate, but important roles in pneumonia, such as neutrophil recruitment and tissue protection [19–23]. But to date, the role of localized IL-11 in acute pulmonary infections, such as pneumonia, is unknown. Given the previously established role of IL-11 in other inflammatory contexts as well as the known importance of other select IL-6 family members, we investigated the functional contributions of IL-11 in the setting of bacterial pneumonia.

## Materials and methods

### Mouse strains

C57BL/6J mice were purchased from Jackson Laboratories (Bar Harbor, ME). We previously generated EpiRelA^-/-^ (mice with RelA deleted specifically in lung epithelial cells [24]) and MyeloidRelA^-/-^ (mice with RelA deleted specifically in myeloid lineage cells [25]) mice by crossing RelA-floxed mice with NKX2.1-Cre (promotor for NK2 homeobox 1, a marker of lung epithelial cells, driving Cre recombinase expression) or LysM-Cre mice (promotor for the lysozyme 2 gene, expressed in myeloid lineage cells, driving Cre recombinase expression) respectively. Mice were housed in the Boston University animal facility and all animal protocols were approved by the Boston University Institutional Animal Care and Use Committee (IACUC, #PROTO201800710). Studies included both male and female mice, which were between six and twelve weeks old. For EpiRelA^-/-^ and MyeloidRelA^-/-^ experiments, littermate Cre-negative (Cre-) mice were used as controls in all experiments.

### Pneumonia model

Mice were anesthetized with an intraperitoneal (IP) injection of ketamine (50 mg/kg, Zoetis, Kalamazoo MI) and xylazine (5mg/kg, Henry Schein Animal Health, Waltham, MA). Experimental infections were induced by intratracheal (IT) instillation of 50μl saline with approximately 1–2 x 10^6^ CFU *Escherichia coli* (*E*. *coli* serotype 06:K2:H1; ATCC #19138; ATCC, Manassas, VA), or one of two strains of *Streptococcus Pneumoniae (S*. *pneumoniae* serotype 3, ATCC 6303 or serotype 19 EF3030, kindly provided by Dr. Marc Lipsitch, Harvard School of Public Health) into the left bronchus as previously described [19, 26]. We chose *E*. *coli* as our primary pathogen because it is major cause of health care associated pneumonia [27] and is a well-established and tractable animal model of gram-negative bacterial pneumonia [19, 28, 29]. For some experiments (as described in the text), 10μg anti-IL-11 or IgG control (R&D Systems, Minneapolis, MN), or 50ng rmIL-11 (R&D Systems) or PBS control was IT instilled alone or in combination 1–2 x 10^6^ CFU *E*. *coli* in a total volume of 50μl. After the indicated length of infection, the mice were euthanized by isoflurane overdose (Henry Schein Animal Health).

### Lung harvest and bronchoalveolar lavage

Bronchoalveolar lavage (BAL) was performed as previously described [19]. Briefly, the heart-lung block was removed and suspended by the trachea on a blunt catheter. Lungs were serially lavaged with 1ml ice cold PBS for a total of 10ml. Lavage fluid recovered from the first 1ml was centrifuged to remove cells and debris, and supernatant was used for total protein and cytokine determination. Cells from the remaining 9ml of lavageate were combined with those from the initial 1ml wash to assess total and differential cell counts. Lavaged lungs were snap-frozen in liquid nitrogen, and kept at -80°C for future determination of either RNA or cytokines (see below). Total cells from BALF were counted on a Luna *fl* fluorescence cell counter (Logos biosystems, Annandale, VA), differentials were counted manually on Diff-Quick (VWR, Radnor, PA) stained slides after cryocentrifugation (Shandon Cytospin, Thermo Fisher Scientific, Waltham, MA). Total protein in BAL fluid was measured using a Bicinchoninic acid assay (Millipore Sigma, St. Louis, MO).

### LDH assay

LDH activity was measured in BAL fluid using a CytoTox 96 non-radioactive cytotoxicity assay (Promega, Fitchburg, WI), following the manufacturer’s instructions. Colorimetric change was measured at OD_450_ using a BioTex Synergy Lx multiplate reader (BioTex, Houston, TX).

### Cytokine determination

Snap-frozen lungs were homogenized using a Bullet Blender (Next Advance, Averill Park, NY), and total protein was isolated from lungs using protein extraction buffer as described [19]. IL-11 concentrations in lung homogenates and BAL fluid were determined using ELISA (Duoset kit, R&D systems), per the manufacturer’s instructions. G-CSF, CXCL1, CXCL2, CXCL5, CXCL10, TNFα, and IL-6 concentrations in BALF were measured using a mouse magnetic Luminex assay (R&D Systems), read using a LiquiChip 200 workstation (Qiagen, Valencia, CA).

### H&E staining

Prior to fixation, lungs were perfused with 10ml ice cold PBS. The heart-lung block was isolated and lungs were inflated to 20 mmH_2_O with 4% PFA in PBS (Ted Pella, Redding, CA). Fixed lungs were embedded in paraffin, blocks cut into 5μm sections and stained with hematoxylin and eosin [30] (Millipore Sigma). Stained sections were imaged using a Leica DM4B fluorescent microscope with DFC7000 digital camera, and data processed in LAS X imaging software (Leica Microsystems, Wetzlar, Germany). A minimum of 20 random 400x images from each sample were used for injury scoring as described [31]. Briefly, a blinded investigator scored each image based on alveolar neutrophils, interstitial neutrophils, hyaline membrane formation, proteinaceous debris in airspace and alveolar septal thickening. To generate a lung injury score, the sum of the five variables were weighted according to relevance ascribed in [31], and normalized to number of fields evaluated. The injury score is a value between zero and one inclusive.

### Immunoblotting

Snap-frozen lungs were homogenized using a Bullet Blender (Next Advance), and total protein was isolated from lungs using protein extraction buffer as described [19]. Total protein concentrations were determined using a Bicinchoninic acid assay (Millipore Sigma). Equal amounts of protein were loaded onto a NuPAGE 4–12% Bis-Tris gel (Thermo Fisher Scientific), and transferred to Immobilon-P PVDF membrane (Millipore Sigma) using the X-Cell Blot II system. Membranes were probed with rabbit anti-Y705-pSTAT3 or total STAT3 polyclonal antibody (Cell Signaling Technology, Danvers MA), followed by anti-rabbit IgG-HRP (Cell Signaling Technology) and developed with ECLPlus (GE Healthcare, Chicaco, IL) before exposing to film (GE Healthcare).

### Lung digestion and flow cytometry cell sorting

Left lobes were digested as previously described [32]. Briefly, after perfusion with 10ml ice-cold HBSS (Thermo Fisher Scientific), the heart-lung block was removed and the lungs were serially lavaged with 10ml DPBS (Thermo Fisher Scientific), followed by 1ml RPMI 1640. The lungs were inflated with 1ml digestion solution (RPMI media with 4.5U elastase [Roche, Basel, Switzerland], 10% dextran [Millipore Sigma], and 100U/ml DNase [Qiagen]), followed by 0.5 ml 1% low melting temperature agarose (Millipore Sigma). After cooling on ice to solidify agarose, the left lobe was separated from the heart-lung block and incubated at 37°C for one hour in additional digestion solution with gentle rotation. The tissue was gently minced and incubated in RPMI 1640 medium containing 50% FBS for an additional 15 min. Suspensions were the sequentially filtered through 100, 70 and 40μm filters (Thermo Fisher Scientific). Lung single-cell suspensions were subjected to FACS using a FACSARIAII cell sorter (BD Biosystems, Franklin Lakes, NJ). Dead cells were excluded using 7-aminoactinomycin D (7-AAD, BD Biosciences). Epithelial cells (CD45^-^/Ly6G^-^/EpCam^+^), neutrophils (CD45^+^/Ly6G^+^/EpCam^-^), and non-neutrophil leukocytes (CD45^+^/Ly6G^-^/EpCam^-^), were sorted using the following antibodies from eBioscience—CD45-FITC (Clone 30-F11), Ly6G-APC-Cy7 (clone 1A8), and EpCam-APC (CD326, clone G8.8). Cells were sorted into PBS with 1% BSA, immediately centrifuged at 300G and resuspended in RNAprotect (Qiagen) to preserve RNA integrity prior to subsequent analyses.

### RNA isolation and qRT-PCR

For flow cytometry sorted cells, cells were disrupted using a QIAshredder column (Qiagen), after which RNA was isolated using a RNeasy micro kit (Qiagen) following the manufacturer’s instructions. For whole lobe RNA, left lobes were homogenized using a Bullet Blender (Next Advance, Averill Park, NY). RNA was isolated from homogenates using the RNeasy mini kit (Qiagen). qRT-PCR was performed using a QuantStudio 3 Real-Time PCR System (Thermo Fisher Scientific) and the TaqMan RNA-to Ct 1-step kit (Thermo Fisher Scientific). Primer and FAM-labeled probe sets for *IL-11* and *IL-11R*α (Thermo Fisher Scientific) were multiplexed with Eukaryotic 18S rRNA Endogenous Control (Life Technologies, VIC labeled). Expression values are presented as fold induction and normalized to 18s rRNA as previously described [33].

### Statistical analysis

Statistical analyses were performed using Graph Pad Prism (Version 8, GraphPad, La Jolla, CA). Data are presented as mean ± SEM. Data normality was analyzed using a Shapiro-Wilk test, and non-normally distributed data were analyzed using a non-parametric test for significance when possible, or log transformed prior to further statistical analysis. Comparisons were considered significant when p<0.05. Specific tests used and sample sizes for each experimental group are described in figure legends.

## Results

### Basal IL-11 expression is elevated during normal homeostasis

**Fig 1:**
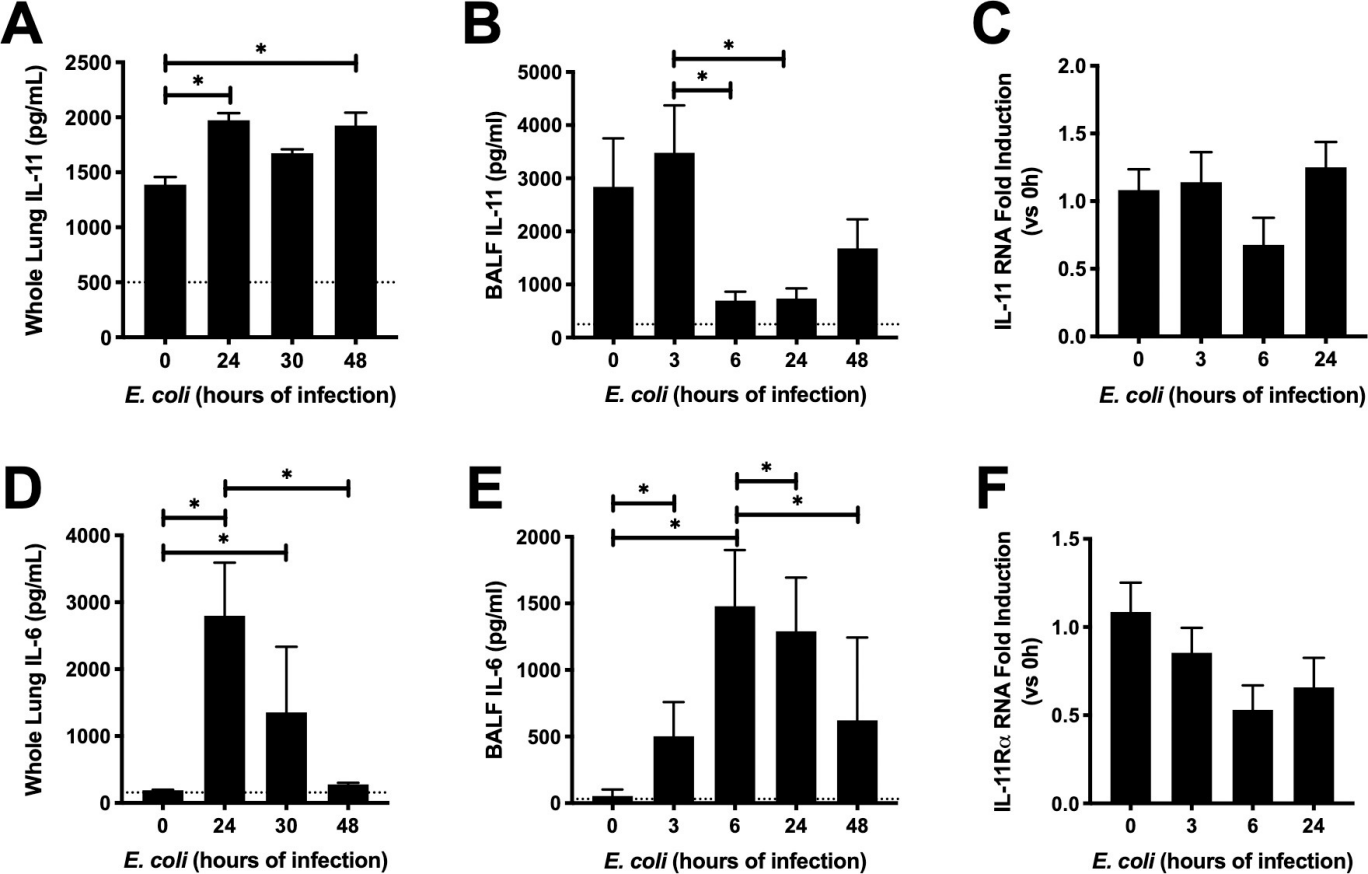
Temporal regulation of IL-11 in the lungs during pneumonia. Mice were infected with approximately 10^6^ CFU *E*. *coli* for the time indicated. Whole lungs were harvested and analyzed for A) IL-11 protein, C) IL-11 RNA, D) IL-6 protein and F) IL-11Rα RNA. BALF was collected and B) IL-11 and E) IL-6 protein were measured. RNA data were tested by ordinary one-way ANOVA and Tukey’s correction for multiple comparisons (N = 4–9). IL-11 and IL-11Rα RNA levels were not significantly changed. BALF and whole lung cytokine data were tested with Kruskal-Wallis one-way ANOVA and Dunn’s correction for multiple comparisons (N = 7–19). * P<0.05 Dotted line indicated lowest level of detection, where applicable.

We have previously shown that IL-11 mRNA is significantly but modestly induced in the lung during *E*. *coli* pneumonia after 24 hours of infection [19]. Given the important role other gp130-signaling cytokines play in pneumonia, along with IL-11’s established roles in other inflammatory settings, we sought to understand the role of IL-11 in pneumonia. We began by establishing how IL-11 changes temporally during infection (Fig 1). Interestingly, whole lung IL-11 content was maintained at relatively high concentrations in uninfected mice, and were elevated even further as a consequence of pneumonia (Fig 1A). This expression pattern contrasts that of the related cytokine IL-6, which was lowly expressed at baseline, and robustly increased during infection, before returning to baseline by 48h (Fig 1D and 1E). Indeed, basal levels of IL-11 were approximately 10-fold higher than those observed for IL-6 in our present study, and 10-100-fold higher than levels of other IL-6 family cytokines published elsewhere [21, 22]. In contrast to our findings in lung tissue, levels of IL-11 protein in BALF decreased after 6h of infection, recovering to baseline levels by 48h (Fig 1B). Moreover, we failed to detect an increase in IL-11 mRNA following *E*. *coli* infection (Fig 1C), which was surprising given our prior observation of modest yet statistically significant induction, albeit in mice bred on a different (mixed) genetic background [19]. Given the subtlety of IL-11 dynamics combined with its particularly high baseline concentrations, we also considered the possibility that IL-11 responsiveness may be dictated by changes in its specific receptor IL-11Rα. However, IL-11Rα mRNA levels were unchanged during pneumonia (Fig 1F), suggesting that IL-11 signaling capacity is unlikely a function of receptor density.

### IL-11 expression is independent of NF-κB and produced by epithelial cells

**Fig 2:**
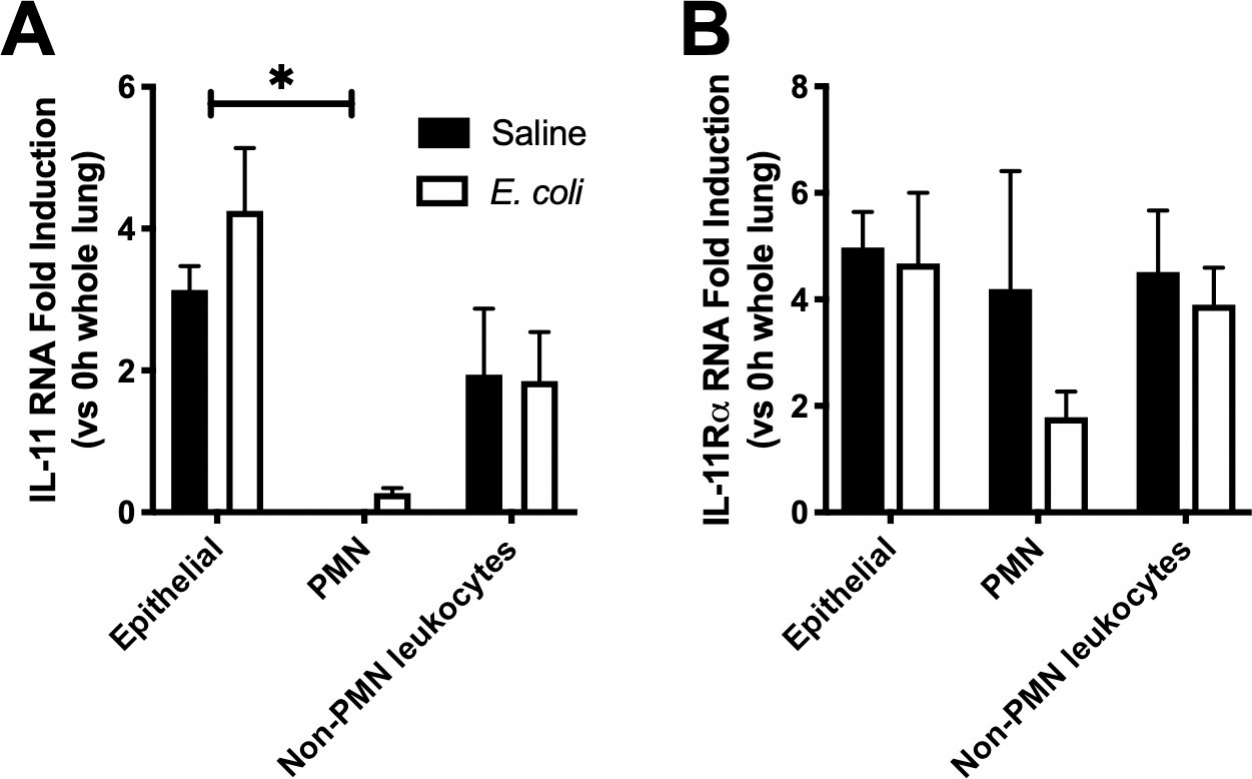
Cellular source of IL-11 and IL-11Rα. Left lung lobes were harvested from uninfected mice and mice challenged for 24 hours with *E*. *coli* or saline control. Lungs were digested to single cell suspension, after which lung epithelial cells (CD45^-^/Ly6G^-^/EpCam^+^), neutrophils (CD45^+^/Ly6G^+^/EpCam^-^), and non-neutrophil leukocytes (CD45^+^/Ly6G^-^/EpCam^-^), were isolated by FACS. RNA was isolated from cells and analyzed for A) IL-11 and B) IL-11Rα. Significance was tested with 2-way ANOVA, with Tukey’s correction for multiple comparisons. * P<0.05. N = 4–5.

Next, we examined the cellular sources of IL-11 and IL-11Rα. Others have shown the majority of IL-11 to be produced by leukocytes, fibroblasts and epithelial cells to varying degrees in a given organ system [34]. In the early phase of pneumonia, cytokine mediators are largely produced by resident immune cells (primarily macrophages), epithelial cells, and subsequently recruited leukocytes (primarily neutrophils), often involving the prototypical inflammatory transcription factor NF-**κ**B RelA (p65) [3]. In order to explore IL-11 production in these cell types, we sorted epithelial cells (CD45^-^/Ly6G^-^/EpCam^+^), neutrophils (CD45^+^/Ly6G^+^/EpCam^-^), and non-neutrophil leukocytes (CD45^+^/Ly6G^-^/EpCam^-^) from lung single-cell suspensions generated from mice treated with saline or *E*. *coli*. When normalized to values collected from uninfected lung homogenates, IL-11 transcription was significantly higher in epithelial cells compared to neutrophils and other leukocytes, regardless of infection (Fig 2A). This suggests epithelial cells are a prominent source of IL-11 in both the absence and presence of pneumonia. In contrast, IL-11Rα mRNA levels were uniform across all cell types examined (Fig 2B). This is consistent with our whole lung findings, (Fig 1D) where IL-11Rα remained unchanged during infection.

**Fig 3:**
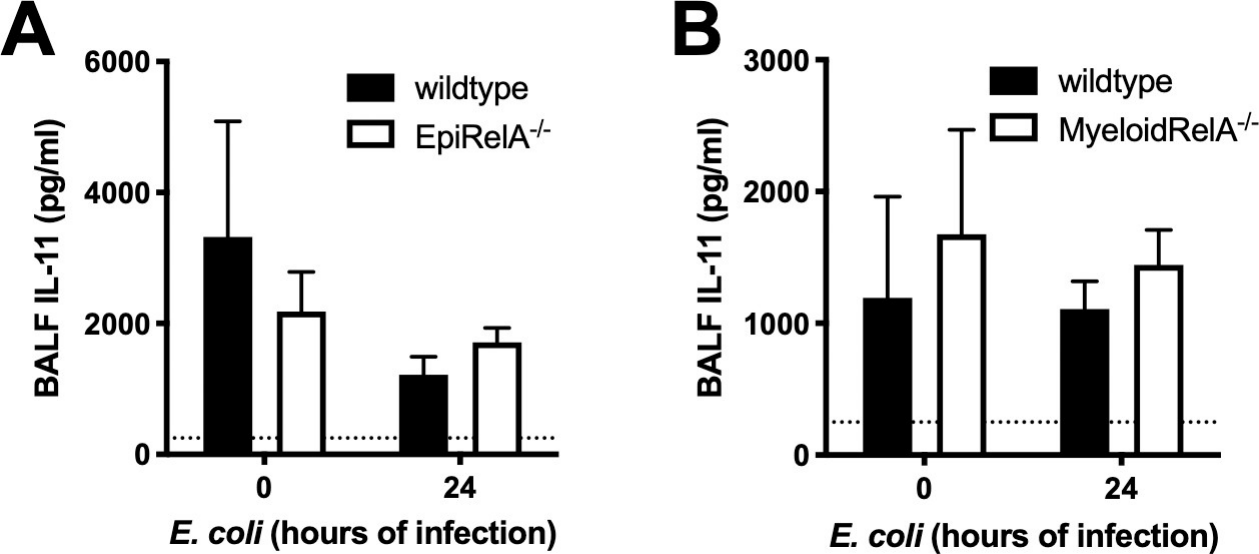
IL-11 production is not NFκB dependent. (A) EpiRelA^-/-^ or wildtype mice or (B) MyeloidRelA^-/-^ or wildtype mice were infected with *E*. *coli* for 24h and BALF IL-11 was measured. Significance was evaluated by two-way ANOVA with Sidak correction. Dotted line represents lowest limit of detection. (N = 3–8).

Early work involving IL-11 demonstrated that *in vitro* IL-1β administration results in production of IL-11 [35]. We have previously shown that production of IL-1β and other cytokines is dependent on NFκB signaling in the lung during pneumonia [24, 36]. We investigated whether production of IL-11 is similarly NFκB-dependent using mice with NFκB RelA selectively mutated in cells of either epithelial (Fig 3A) or myeloid (Fig 3B) lineage. Consistent with our results using WT C57BL/6J mice (Fig 1), infection had no influence on BALF IL-11 concentrations, nor did NFκB targeting in either cell type (Fig 3), suggesting that the relatively high abundance of this cytokine is maintained by alternative signaling requirements and/or cell types other than those of myeloid or epithelial lineage.

### Neutralization of IL-11 results in worsening alveolar protein leak, and impairs neutrophil recruitment

**Fig 4:**
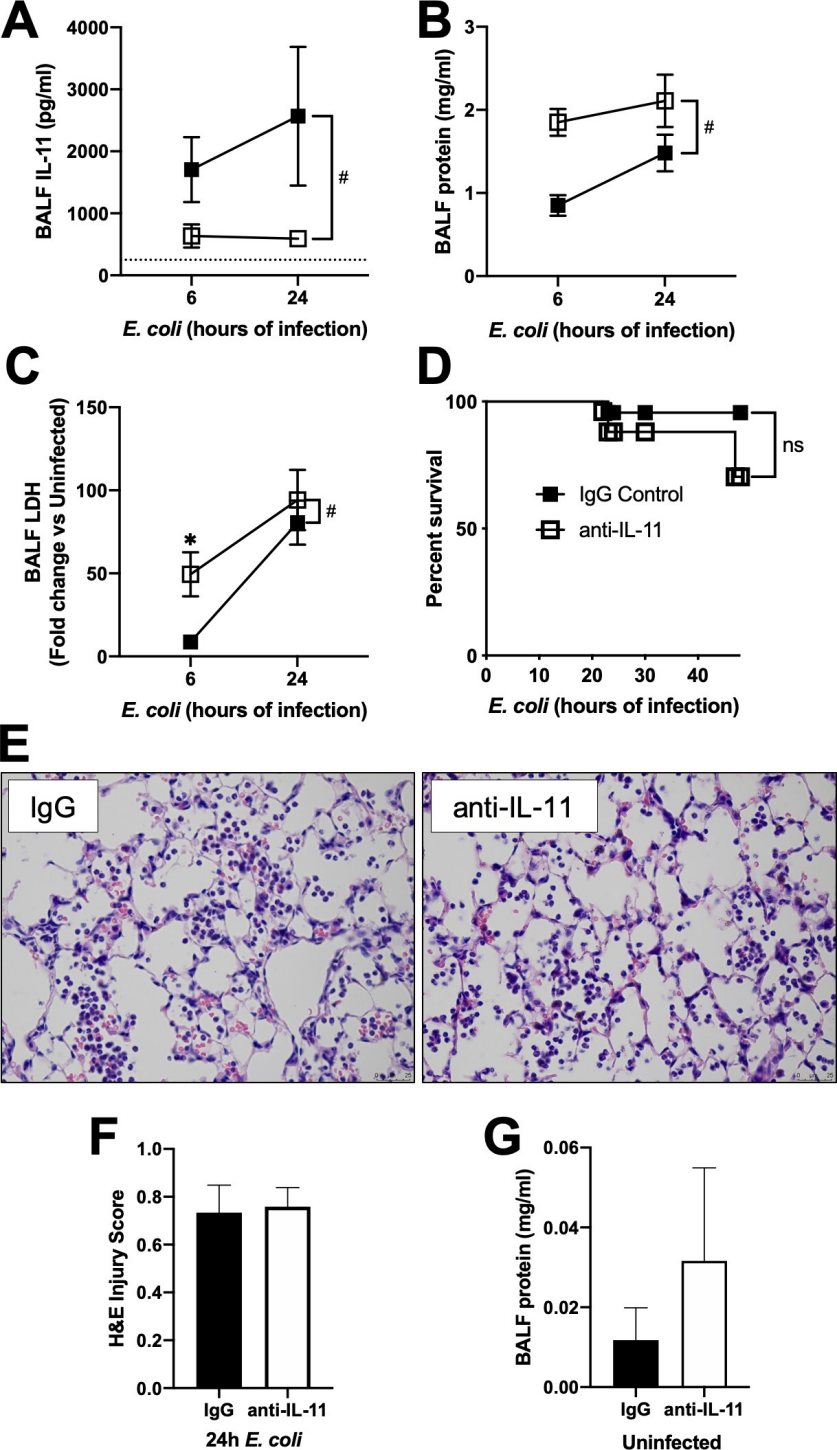
IL-11 neutralization affects lung injury. Mice were infected with *E*. *coli* plus IgG control or anti IL-11 neutralizing antibody. BALF was collected at the time indicated and analyzed for A) IL-11 protein, B) total protein, and C) LDH activity. N = 4–8. Significance was tested with two-way ANOVA and Sidak’s correction for multiple comparisons. # significant for treatment by ANOVA, * p<0.05. D) Mouse survival. N = 23–25. Survival significance was determined by Log-rank test. ns not significant. Lungs were fixed with PFA, embedded in paraffin, and 5μm sections were stained with H&E. E) representative images of control IgG and anti-IL-11 treated lungs. Bar 25 μm. F) quantification of lung injury. N = 5–6. Significance was tested with a Student’s unpaired t-test. In a separate experiment, mice were treated with IgG or anti-IL-11 in the absence of infection for 24h and G) total BALF protein measured. N = 5–6. Significance tested with a Student’s unpaired t-test.

Since IL-11 levels are maintained at relatively high levels at baseline, we utilized neutralizing antibodies to assess the roles of this cytokine during pneumonia. Treatment with a neutralizing IL-11 antibody at the time of infection significantly decreased BALF IL-11 protein detection (Fig 4A). IL-11 neutralization increased BALF total protein levels, indicating an increase in proteinaceous edema (Fig 4B). Exaggerated edema following IL-11 blockade was also associated with increased cell death based on elevated LDH activity in BALF (Fig 4C). However, we did not observe histological evidence of increased lung injury in mice treated with anti-IL-11 (Fig 4E and 4F), nor was there an effect on short-term survival (Fig 4D). Finally, we evaluated the effect of IL-11 neutralization in the absence of infection, and did not see a significant difference in BALF protein (Fig 4G). Overall, these data suggest that while neutralization of IL-11 promotes lung injury during pneumonia, albeit relatively mildly (based on histological examination), baseline homeostatic levels of IL-11 do not appear to affect tissue integrity.

**Fig 5:**
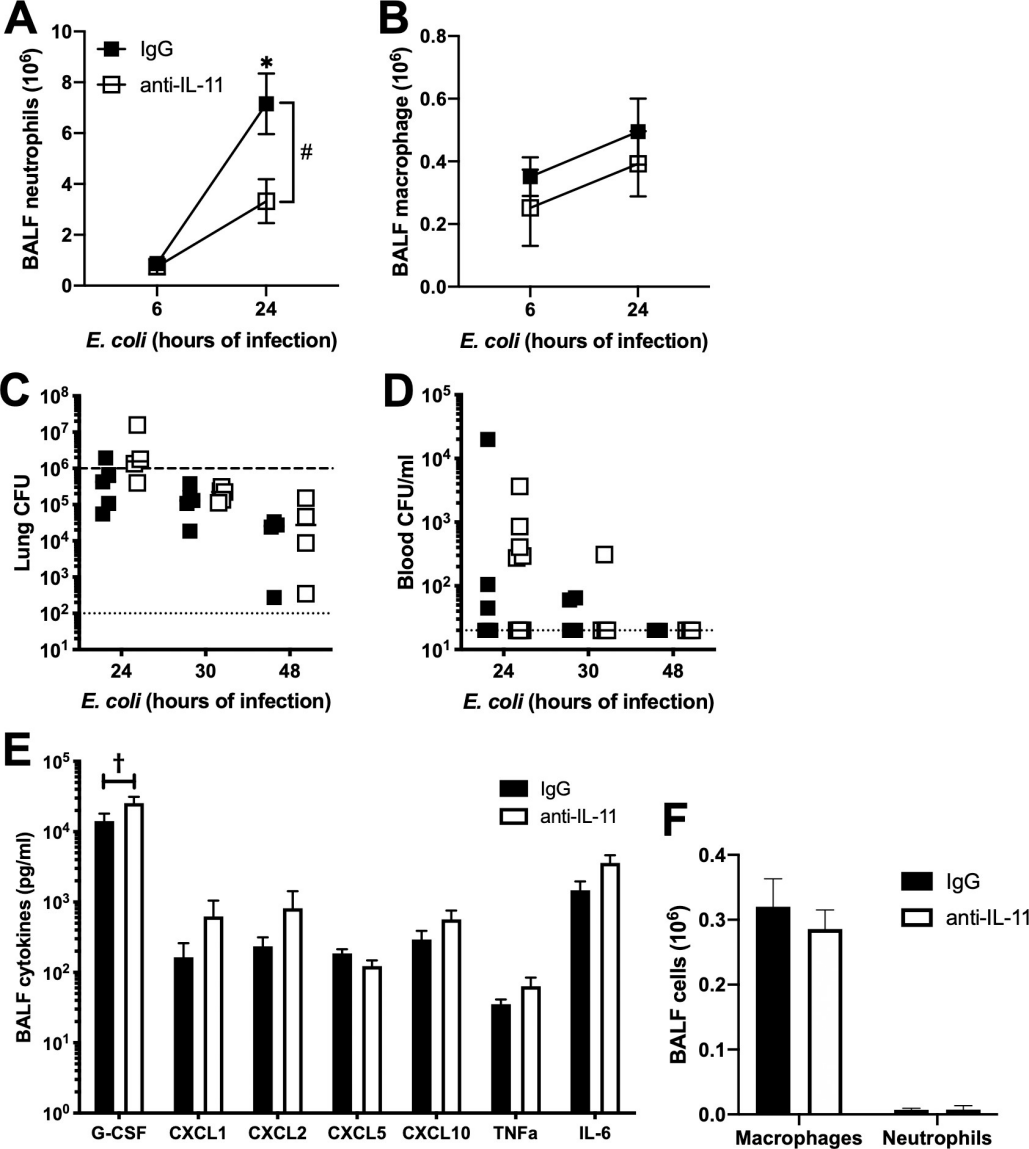
IL-11 neutralization affects cellular recruitment. Mice were infected with *E*. *coli* plus IgG control or anti IL-11 neutralizing antibody for the time indicated. BAL cells were collected and analyzed for A) neutrophil and B) macrophage number. N = 6–8. Significance was tested with two-way ANOVA and Sidak’s correction for multiple comparisons. # significant for treatment by ANOVA, * p<0.05. BALF fluid from 24h infections were also analyzed for E) cytokines, and significance was determined by multiple t tests. N = 6. † FDR<0.05. C) whole lung and D) blood were analyzed at the indicated time for bacterial burden. N = 4–13. In a separate experiment, mice were treated with IgG or anti IL-11 neutralizing antibody without infection for 24h and BAL cells were collected and analyzed for F) total macrophage and neutrophil number. N = 5–6. Significance was tested with two-way ANOVA and Sidak’s correction for multiple comparisons.

We next looked to see whether neutralization of IL-11 affected lung cellularity during pneumonia (Fig 5). Neutralization of IL-11 decreased neutrophil recruitment, but did not alter airspace macrophage numbers following 24h of infection (Fig 5A and 5B). This decrease in neutrophils was not accompanied by a deficit in bacterial clearance (Fig 5C), bacteremia (Fig 5D) or survival (Fig 4D). We then measured cytokine production to evaluate the basis for change in neutrophil recruitment. Unlike the related IL-6 family member Oncostatin M (OSM), which we have previously shown to affect neutrophil recruitment in a CXCL5-dependent manner [22], neutralization of IL-11 does not change CXCL5 levels in BALF. In addition, there is no significant difference in other neutrophil-related chemokines and cytokines (Fig 5E). Curiously, G-CSF was elevated in association with reduced neutrophilia, but this is more likely a compensatory consequence of reduced neutrophils rather than a cause. Finally, neutralization of IL-11 in the absence of infection had no impact on alveolar cellularity (Fig 5F).

**Fig 6:**
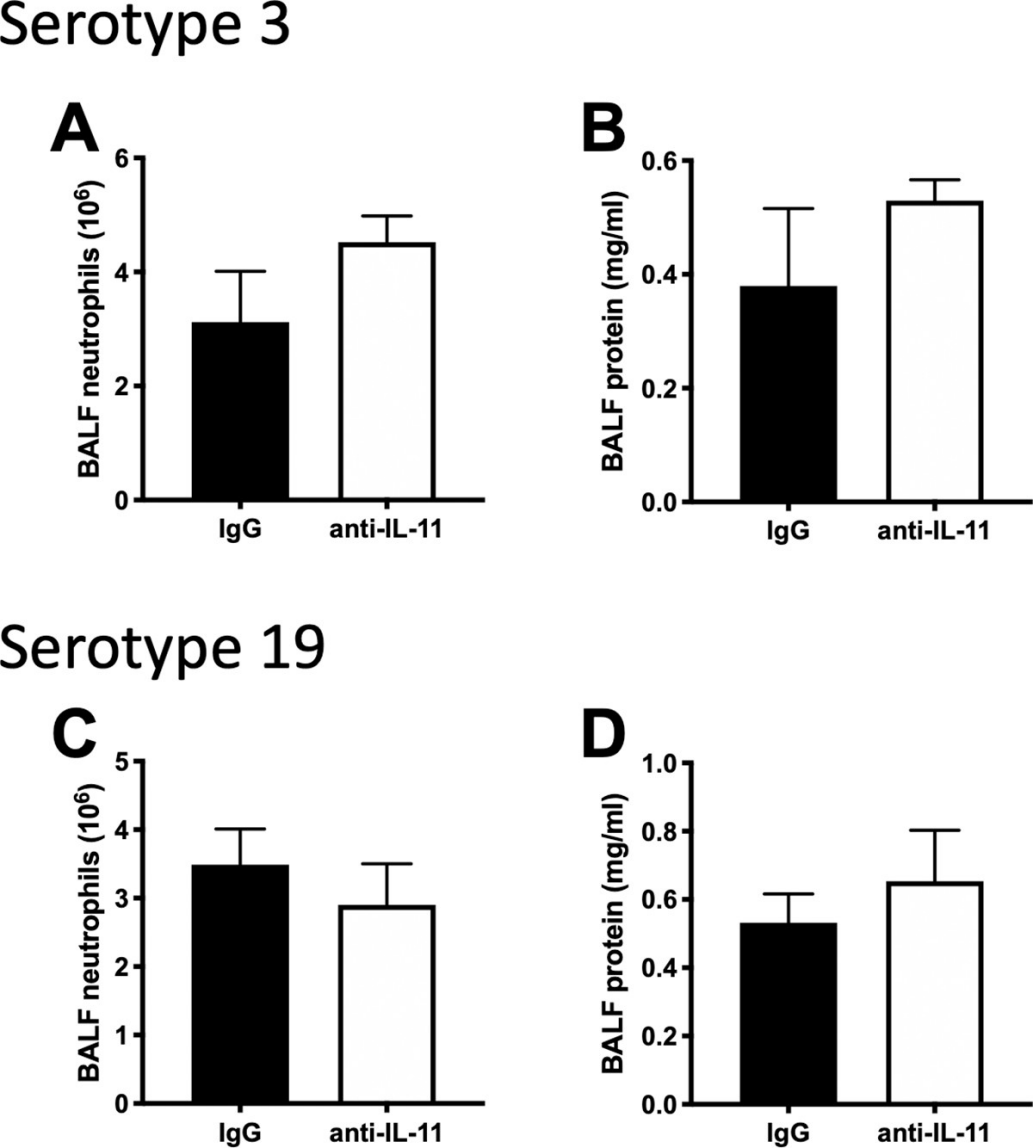
IL-11 neutralization has little effect on pneumococcal pneumonia. Mice were infected with *S*. *pneumoniae* either Serotype 3 (A-B) or 19 (C-D) in the presence of anti-IL-11 or IgG control for 24h. BALF was collected and analyzed for A, C) neutrophils and B, D) protein. Significance was tested with T-test. N = 3–5.

We then examined whether or not a similar effect could be detected in response to pneumonia caused by a gram-positive bacterium, *S*. *pneumoniae* (Fig 6). Neutralization of IL-11 did not alter BALF protein levels (Fig 6B and 6D) or neutrophil recruitment (Fig 6A and 6C) during *S*. *pneumoniae* pneumonia, with either a high (serotype 3, Fig 6A and 6B) or low (serotype 19, Fig 6C and 6D) virulence serotype. In contrast to *E*. *coli* pneumonia, the pneumococcal infections caused very little pulmonary edema (Figs 4B, 6B and 6D).

### IL-11 administration does not affect lung injury or cellularity during pneumonia

**Fig 7:**
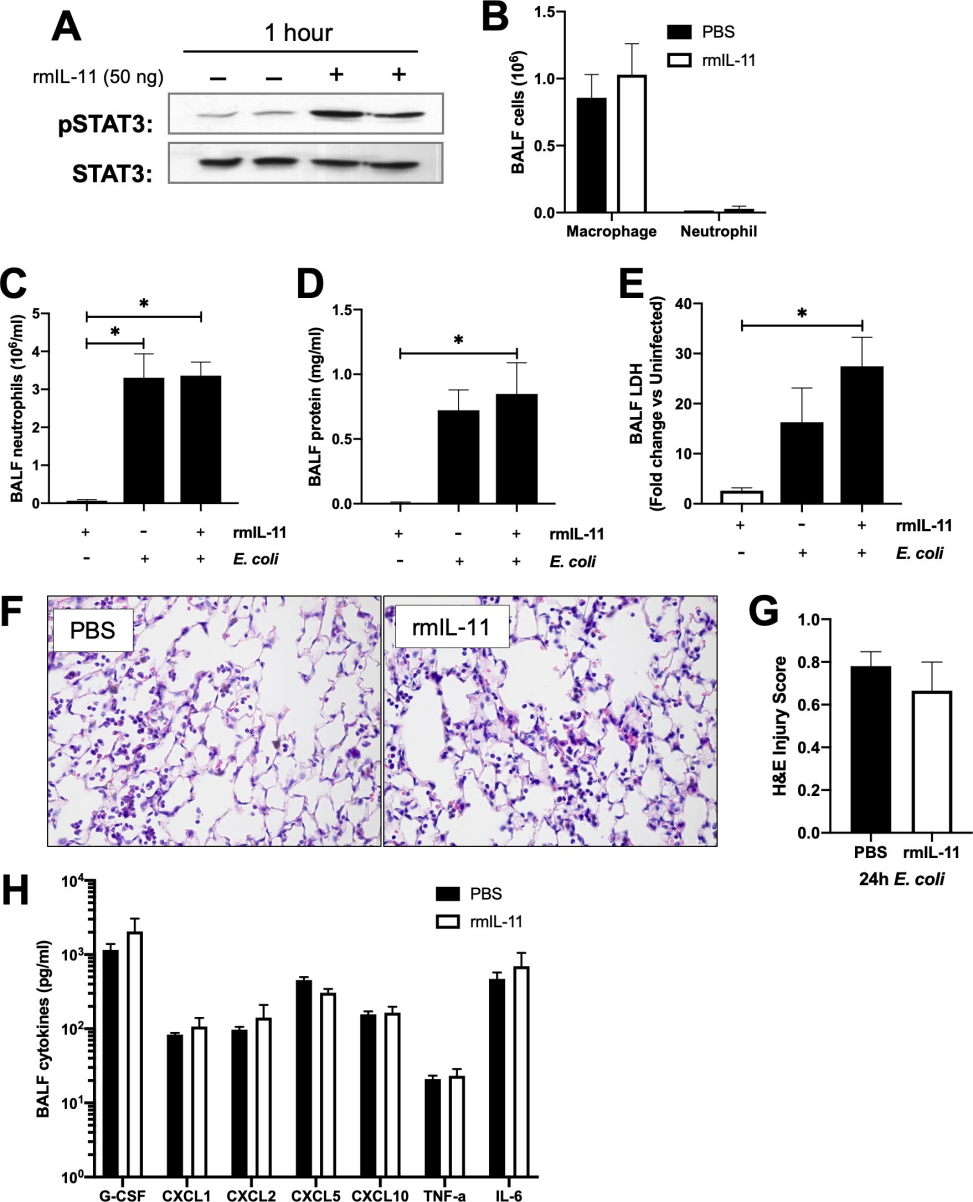
Exogenous IL-11 activated STAT3, but does not affect pneumonia outcomes. Mice were treated with 50ng rmIL-11 or PBS control for various times. A) Representative immunoblots of Y705-STAT3 (pSTAT3) and total STAT3 protein content in lung homogenates after 1h rmIL-11. B) BALF was collected after 6h of IL-11 in the absence of infection, and analyzed for cellularity. BALF was collected after 24h rmIL-11 in the absence or presence of *E*. *coli* and analyzed for C) neutrophils, D) protein E) LDH, and H) cytokines. Significance was tested with one-way ANOVA and Tukey’s correction for multiple comparisons * p<0.05. In a separate experiment, lungs were fixed with PFA, embedded in paraffin, and 5μm sections were analyzed for F-G) H&E staining. Images are representative of control IgG and rmIL-11 treated lungs. Bar indicates 25μm. Significance was tested with a Student’s unpaired t-test.

Several studies examining IL-11 in systemic infections have demonstrated that rmIL-11 has anti-inflammatory roles [10, 11]. This effect is mediated in some cases through modulation of JAK-STAT pathways. We have shown that other members of the IL-6 cytokine family exert their function through activation of the transcription factor STAT3 [19, 20, 22, 23]. We investigated whether localized rmIL-11 has an effect on STAT3 activation. In our model, addition of exogenous IL-11 in the absence of infection was sufficient to increase levels of active Y705-phosphorylated STAT3 (pSTAT3), in whole lung tissue (Fig 7A), but had no effect on BALF cellularity (Fig 7B). Furthermore, the rmIL-11 treatment during *E*. *coli* pneumonia did not affect neutrophil recruitment (Fig 7C) or inflammation or injury as measured by BALF protein (Fig 7D), LDH activity (Fig 7E), lung histology (Fig 7F and 7G), or cytokine production (Fig 7H). Overall, these data suggest that the addition of localized rmIL-11 at the site of pneumonia does not significantly impact pneumonia outcome.

## Discussion/Conclusions

Several studies suggest that systemic IL-11 plays an important role in regulating tissue injury and inflammation during lung infections [10, 11, 17, 37]. Given that many IL-6 family cytokines are induced in murine models of bacterial pneumonia, and modulation of these cytokines results in disparate effects on host defense and resilience to lung injury [20–22], we sought to determine functional contributions of IL-11 in response to lung infection. Unlike its IL-6 family counterparts studied previously, we found that IL-11 levels are temporarily but significantly reduced in the alveolar lining fluid in response to infection. In the lung parenchyma, IL-11 levels are maintained at high levels prior to infection, and are modestly yet significantly increased thereafter. Paradoxically, neutralization of IL-11 exaggerated alveolar edema whilst reducing neutrophil accumulation in the airspaces, whereas the addition of supplemental recombinant IL-11 protein had no detectable biologic effect in acute pneumonia. We conclude from these results, however, that while IL-11 may impact the balance of acute pulmonary inflammation to some extent, such effects are relatively subtle, especially compared to those identified for other IL-6 family members under similar conditions [20–22].

The lack of substantial IL-11 induction in the pneumonic airspaces contrasts the dynamic nature of IL-6, which is maintained at low levels at baseline and then strongly induced with pneumonia. Absence of a detectable IL-11 mRNA response also contradicts a previous study by our own group in which we detected minor induction of IL-11 mRNA in lung homogenates by 24h after intratracheal *E*. *coli* inoculation [19]. We speculate that this discrepancy is due to variations in the magnitude and/or timing of IL-11 induction between different mouse strains, as the prior study was performed using mice bred on a mixed genetic background, and our current study included mice on an inbred C57BL/6J background. Yet, even in our prior study the IL-11 mRNA induction was extremely low compared to other IL-6 family cytokines in the same samples, consistent with our reduced ability to detect it here. Moreover, our unpublished RNASeq results indicate extremely low counts for IL-11, suggesting either a low transcription capacity or high lability, either of which could impact the interpretation of fold-change measurements. Finally, alterations in IL-11 protein levels that were seen with infection could be the result of post-transcriptional regulation, rather than alteration in IL-11 mRNA levels.

We aimed to determine cellular sources of lung IL-11 using sorted cells from single cell suspension. In our model system, it appears that epithelial cells are a predominant source of IL-11, unlike in other organ systems, where IL-11 is produced in non-epithelial cell types, such as fibroblasts, hepatocytes and chondrocytes [34]. Earlier studies also demonstrated that *in vitro*, IL-11 can be produced by the human lung stromal cell lines MRC-5 and A549 [17, 38]. In addition, we saw a trend toward higher levels of IL-11 RNA in non-neutrophil leukocytes, which is consistent with previous data showing that IL-11 is produced by lung macrophages during TB infection [39]. Therefore, while we demonstrated epithelial cells as a primary source of IL-11 in our model system, it is possible that in the lung, non-epithelial cells could be an additional source of IL-11, and this may be dictated by the type and duration of infectious stimulus. We previously demonstrated that another IL-6 family member, LIF is also produced selectively in epithelial cells [23]. However, unlike LIF, IL-11 is not dynamically regulated at the RNA level in pneumonia, suggesting an alternate regulatory method. This is supported by the fact that while LIF production is dependent on NFκB-RelA in myeloid lineage cells, IL-11 production is maintained independently of NF-κB.

Several prior studies demonstrated that modulation of systemic IL-11 can affect outcomes during systemic infections [10–12]. Intravenous administration of IL-11 reduced tissue inflammation and mortality in neutropenic rats with systemic *P*. *aeruginosa* infection [11], whereas intraperitoneal IL-11 treatment reduced cytokine expression, neutrophil accumulation, and pulmonary edema in mice challenged with endotoxemia [10]. On the other hand, administration of neutralizing anti-IL-11 antibody during a systemic *L*. *monocytogenes* infection impaired bacterial clearance [12]. In several studies, exogenous administration of IL-11 resulted in abrogation of inflammation- or infection-related TNFα increase [8, 9, 40, 41]. However, administration of IL-11 directly into the alveolar space in these studies did not result in changes in TNF-α. In the context of these prior studies, our current data suggests that IL-11 in the airspaces may have a subtler impact on acute inflammation than that present in extra-pulmonary sites.

In a mouse model of tuberculosis, IL-11 was increased during infection, and IL-11 inhibition decreased early neutrophil influx and TB disease severity [37, 42]. Here we also demonstrate that neutralization of IL-11 decreases neutrophil accumulation, but this outcome was not associated with changes in bacterial clearance. While it is possible that neutrophil recruitment remained sufficient for maximal defense following IL-11 blockade in *E*. *coli*-challenged mice, it is also possible, if not likely, that the impact of IL-11 varies across different experimental circumstances. This is certainly the case here, where *S*. *pneumoniae*-challenged mice exhibited no effect of IL-11 neutralization during pneumonia.

Although total IL-11 mRNA does not change in infection, alveolar IL-11 decreases by 6 hours of infection, recovering to baseline levels by 48h. In the absence of infection, our results suggest that these initially high levels of baseline IL-11 do not affect tissue injury, at least based on alveolar protein content. Yet, it is possible that maintaining IL-11 homeostasis precedes a favorable outcome in response to infection. In support of this, we detected increases in both proteinaceous edema and cell death during pneumonia in mice administered anti-IL-11. However, no changes were detected when supplementary rmIL-11 was provided, perhaps due to the sufficiently high IL-11 concentrations both before and during pneumonia. Neutralization of IL-11 also led to decreased neutrophil infiltration, however the mechanism of this decrease is unclear. We did not see a change in neutrophil chemokines, or other cytokines known to be associated with neutrophil recruitment (Fig 5C).

Here we investigated the regulation and roles of lung IL-11 during acute bacterial pneumonia. We demonstrate that IL-11 is maintained at relatively high concentrations, which are only slightly altered by infection. Although neutralization of existing IL-11 content altered edema formation and neutrophil accumulation, albeit to opposite degrees, cytokine blockade had no influence on anti-bacterial defense. In addition, exogenous IL-11 delivery had no detectable influence on pneumonia outcome. Together, these data suggest that IL-11, unlike related cytokines such as IL-6, LIF, and OSM, does not exhibit a prominent role in immune resistance or tissue resistance in the setting of acute lower respiratory infection. However, the biological consequences of IL-11 blockade observed here, while relatively modest, reveal a potential avenue of future investigations focused on mechanisms of injury and inflammation in the lungs.
